# Emergency rollout and conversion procedures during the three-arm robotic open-thoracotomy-view approach

**DOI:** 10.1093/icvts/ivab336

**Published:** 2021-11-29

**Authors:** Noriaki Sakakura, Takeo Nakada, Suguru Shirai, Hirotomo Takahara, Ayumi Suzuki, Yusuke Takahashi, Hiroaki Kuroda

**Affiliations:** Department of Thoracic Surgery, Aichi Cancer Center Hospital, Nagoya, Japan

**Keywords:** Robotic lung resection, Open-thoracotomy-view approach, Vertical port placement, Confronting monitors, Emergency rollout and conversion procedures

## Abstract

**OBJECTIVES:**

To conduct robotic lung resections (RLRs) with views similar to those in open-thoracotomy surgery (OTS), we adopted a vertical port placement and confronting upside-down monitor setting: the robotic open-thoracotomy-view approach (OTVA). We herein discuss the procedures for emergency rollout and conversion from the robotic OTVA to OTS or video-assisted thoracoscopic surgery (VATS).

**METHODS:**

We retrospectively reviewed the cases of 88 patients who underwent RLR with three-arm OTVA using the da Vinci Xi Surgical System between February 2019 and July 2021. Robotic ports were vertically placed along the axillary line, and 2 confronting monitors and 2 assistants were positioned on each side of the patient. Three possible conversions were prepared: (i) emergency thoracotomy using an incision along the ribs in a critical situation, (ii) cool conversion using vertical incision thoracotomy in a calmer condition and (iii) conversion to confronting VATS. All staff involved in the surgery repeatedly rehearsed the emergency rollout in practice.

**RESULTS:**

No emergent or cool conversion to OTS occurred. Two patients (2.3%) experienced confronting VATS conversions. One patient underwent an urgent conversion for a moderate haemorrhage from a pulmonary artery branch during left upper lobectomy in the introduction phase. Another patient underwent a calmer conversion during an extended RS6 + S10a segmentectomy, where staples could not be inserted appropriately due to lung lacerations. In all patients, postoperative courses were uneventful.

**CONCLUSIONS:**

The OTVA setting is a possible option for RLRs. This report describes the emergent rollout and subsequent conversion procedures for this method.

**Figure:**
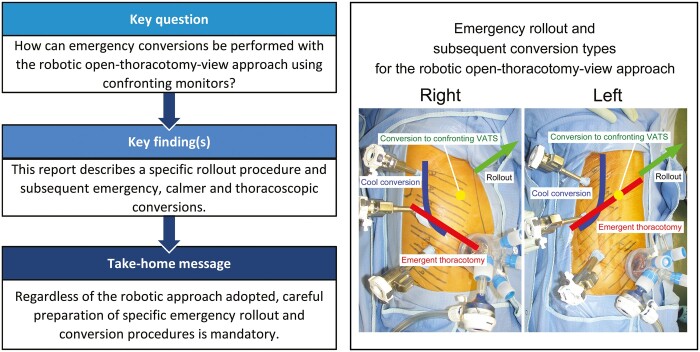

## INTRODUCTION

We recently reported the ‘open-thoracotomy-view approach (OTVA)’ using vertical port placement and confronting upside-down (CUD) monitor settings to conduct robotic lung resections (RLRs) with views similar to those in open-thoracotomy surgery (OTS) [1]. After this report, we received several inquiries about the emergency rollout and thoracotomy conversions for this setting. Emergency rollouts and conversions should be considered for handling serious unexpected events in RLRs, such as massive haemorrhages. World experts have already reported this issue rigorously [2, 3]. Because surgical views and settings in OTVA are different from those in the well-established worldwide conventional look-up view approach, there are some specific considerations to perform this method. To promote robotic OTVA safely, we have devised and repeatedly practiced emergency rollout and conversion procedures in daily practice. Although our experience is still premature, we herein discuss emergent and cool rollouts and conversion procedures from robotic OTVA to OTS or video-assisted thoracoscopic surgery (VATS).

## PATIENTS AND METHODS

### Ethics statement

The institutional review board of Aichi Cancer Center Hospital approved the study (# 2020-1-232). Each patient provided informed consent for the use of clinical data.

### Patients

We began to perform robot-assisted thoracoscopic surgery (RATS) at a full scale in February 2019 after insurance coverage for RATS was initiated in April 2018 in Japan. Of the 109 patients who underwent RATS in our department from February 2019 to July 2021, we retrospectively reviewed 88 consecutive patients who underwent RLRs using the three-arm OTVA with the CUD monitor setting (Table 1). We excluded 21 patients who underwent RATS by different methods other than three-arm OTVA (3 patients underwent RLR using the conventional look-up view approach, 1 underwent RLR with 1 monitor and four-arm OTVA without CUD monitor and 17 underwent robotic mediastinal tumour resection in the supine position). The procedures were performed for clinical stage I primary lung cancer and lesions that were strongly suspected early-stage lung cancer based on the eighth tumour–node–metastasis classification system, and resectable metastatic or other lung tumours.

**Table 1:** Baseline characteristics of the 88 patients who underwent robotic lung resections using the three-arm, open-thoracotomy-view approach

Variables	Data^a^
Age (median, range; years)	70 (36–86)
Sex	
Male/female	35 (40)/53 (60)
Smoking status	
Never/former or current	48 (55)/40 (45)
Brinkman index (median, range)	0 (0–2000)
Body condition	
Height (mean ± SD, range; cm)	159 ± 9 (143–181)
Weight (mean ± SD, range; kg)	59 ± 12 (37–114)
Body mass index (mean ± SD, range; kg/m^2^)	23 ± 3 (16–35)
Respiratory function	
%VC (mean ± SD, range; % predicted)	101 ± 13 (62–152)
%FEV1 (mean ± SD, range; % predicted)	98 ± 18 (40–172)
%DLCO (mean ± SD, range; % predicted)	107 ± 22 (69–181)
HRCT findings and size	
Pure GGO/partly solid/solid	8 (9)/50 (57)/30 (34)
LD (mean ± SD, range; cm)	2.1 ± 0.9 (0.7–5.7)
CD (mean ± SD, range; cm)	1.3 ± 0.9 (0–3.7)
MD (mean ± SD, range; cm)	0.8 ± 0.8 (0–3.5)
Preoperative diagnosis	
Lung cancer (c-stage 0/IA1/IA2/IA3/IB)	84 (3/34/30/15/2)
Metastatic lung tumour	3
Lymphoma	1

a Data are presented as indicated or as the number of patients.

CD: consolidation dimension in HRCT lung window; DLCO: diffusing capacity of the lung for carbon monoxide; FEV1: forced expiratory volume in 1 s; GGO: ground-glass opacity; HRCT: high-resolution computed tomography; LD: whole tumour dimension in the HRCT lung window; MD: tumour dimension in HRCT mediastinal window; SD: standard deviation; VC: vital capacity.

### Robotic OTVA setting

In our practice, OTS is routinely performed using the vertical muscle-sparing/splitting thoracotomy (VMST) [4] with the operating surgeon standing on the patient’s right side (i.e. patient’s dorsal side during right-lung surgery and ventral side during left-lung surgery), regardless of the side to be operated on. During VATS, the operating surgeon also stands on the patient’s right side and uses the CUD monitor setting [5]. This setting enables the operating and assisting surgeons to have the same surgical view in OTS and VATS. Based on these backgrounds, we devised our robotic OTVA.

**Figure 1:**
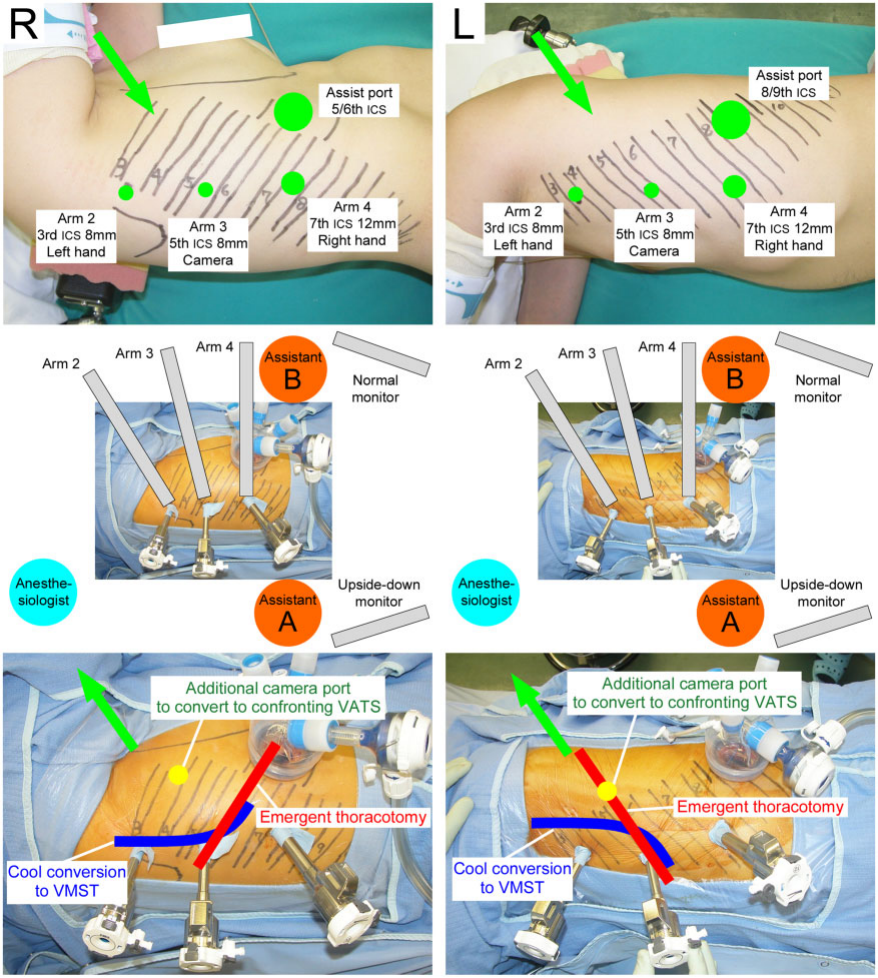
Vertical port placements (top), positions of the robotic arms, 2 assistants and confronting monitors (middle), and possible conversion procedures (bottom) for right-side and left-side surgeries. The lines and numbers drawn on the patient’s body indicate the location of the ribs. The green circles indicate the incision size and intercostal space where each port is placed. Arrows show the roll-in/out directions of the patient cart. The conversion types are as follows: emergency thoracotomy with an incision along the ribs in critical situations (red); cool conversion to vertical muscle-sparing/splitting thoracotomy or axillary incision in calmer conditions (blue); and conversion to confronting video-assisted thoracoscopic surgery by adding a scope port (yellow). The settings for the upper lobes are shown. For middle and lower lobes, the port locations are caudally moved, as described in the text.

The methodology of robotic OTVA has been previously reported in detail [1]. Briefly, the da Vinci Xi^®^ Surgical System (Intuitive Surgical Inc., Sunnyvale, CA, USA) was used. The patient cart was always rolled in from the patient’s left cranial side, regardless of the side to be operated on. In the three-arm setting, the unused arm 1 was pushed towards the cranial side of the patient, arm 2 was positioned at the cranial side of the patient for the console surgeon’s left hand, arm 3 was used for the 30°-robotic endoscopy and arm 4 was positioned on the patient’s caudal side for the console surgeon’s right hand. Robotic ports were vertically placed along the axillary line (Fig. 1, top). For right-side surgeries, 3 robotic ports were placed along the posterior axillary line and 1 assist port was placed on the ventral surface. For left-side surgeries, 3 robotic ports were placed along the anterior axillary line and the assist port was placed on the dorsal surface. For the right upper lobe, an 8-mm robotic port, another 8-mm robotic port and a 12-mm robotic port were placed at the third, fifth and seventh intercostal spaces along the posterior axillary line, respectively and an accessory port was placed on the ventral surface of the fifth or sixth intercostal space; this setup was described as ‘3/5/7/A5 or A6’. Similarly, setups such as ‘3/5/8/A6 or A7’ or ‘4/6/8/A6 or A7’ for the right middle lobe, ‘4/6/8/A6 or A7’ for the right lower lobe, ‘3/5/7/A8 or A9’ for the left upper lobe and ‘3/5/8/A9 or A10’ or ‘4/6/8/A9 or A10’ for the left lower lobe were also used. In these setups, 3 robotic ports were placed around the VMST incision line [4]. Two confronting monitors and 2 assistants were positioned on each side of the patient (Fig. 1, middle). The assistant standing on the patient’s right side (i.e. the patient’s dorsal side for the right-lung surgery or ventral side for the left-lung surgery; assistant A) was mainly responsible for the docking procedure and the exchange of robotic instruments. The assistant standing on the patient’s left side (i.e. the patient’s ventral side for the right-lung surgery or dorsal side for the left-lung surgery; assistant B) directly assisted surgery. The left-side monitor, set up for assistant A, showed the same image as on the surgeon console. The right-side monitor, set up for assistant B, projected the upside-down image of the surgeon console view. These settings provided the console surgeon and the 2 assistants with the same views as our routine OTS and confronting VATS. In this setting, regardless of the side to be operated on, the patient’s craniocaudal axis is aligned with the horizontal direction of the surgeon console monitor, and the cranial and caudal sides of the intrathorax are always displayed on the right and left sides of the surgeon console monitor screen, respectively. An insufflation system (AirSeal^®^ System; ConMed Corporation, Utica, NY, USA) maintained a stable pneumothorax environment because the robotic ports and the target structures are close in this approach.

### Rollout and conversion procedures during the robotic OTVA

**Figure 2:**
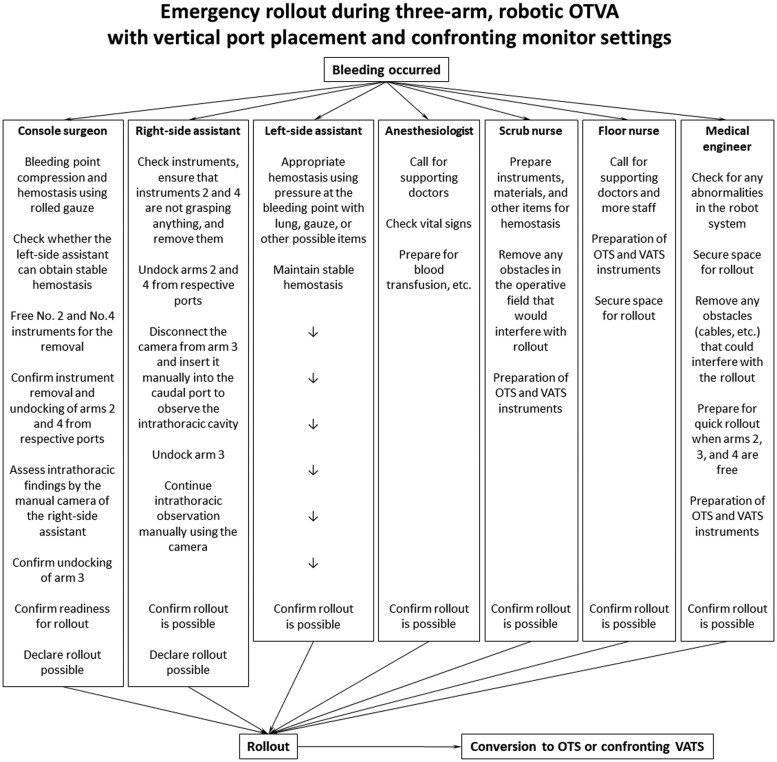
The flow and each staff member’s roles and actions during an emergency rollout for robotic open-thoracotomy-view approach with confronting upside-down monitor settings at our institution. This flowchart was translated to English from the original Japanese version. OTS: open-thoracotomy surgery; VATS: video-assisted thoracoscopic surgery.

The direction and rollout procedure are consistently the same, regardless of the side to be operated on. Figure 1 is our in-hospital document showing our robotic OTVA setting (top, middle) and 3 possible methods of conversion (bottom) in an easy-to-understand manner: (i) emergency thoracotomy conversion with an incision along the ribs in a critical situation, (ii) cool conversion to thoracotomy using VMST or anterior axillary incision in calmer conditions when there is more time and (iii) conversion to regular confronting VATS. Figure 2 is another in-hospital document that summarizes the flow and each staff member’s roles and actions in case of an emergency rollout during RLRs at our institution (revised from the original in Japanese to English). All the staff involved in the RLR surgery confirmed and evaluated these documents, including the console surgeon, right-side assistant surgeon, left-side assistant surgeon, anaesthesiologists, scrub nurses, floor nurses and medical engineers. At our institution, the roll-in/out of the patient cart is performed by medical engineers who are always on standby. The staff members repeatedly practiced emergency rollout procedures together and sometimes rehearsed these procedures in practice assuming that bleeding occurred in patients who were completing RLR uneventfully.

The roles of the operating surgeons in promptly conducting an urgent rollout during OTVA, described in the left 3 columns in Fig. 2, are summarized as follows: (i) the console surgeon and assistants confirm that the instruments of arms 2 and 4 are free (not grasping anything) and assistant A removes these instruments; (ii) assistant A undocks arms 2 and 4 from the respective ports; (iii) assistant A removes the camera from arm 3 and promptly inserts it manually into the caudal port to view the intrathorax; and (iv) arm 3 is undocked, and the patient cart is promptly rolled out. This procedure can be completed in 20 or fewer seconds. Meanwhile, assistant B concentrates on haemostasis. Thereafter, as shown in Fig. 1, emergency thoracotomy or cool conversion to VMST or anterior axillary incision can be selected according to the circumstances. When the situation is not severe, the system can easily be converted into a routine confronting VATS by adding a scope port. As another optional management for a catastrophic situation, a thoracotomy can be performed without rollout only by lifting the 2 caudal arms.

## RESULTS

Table 2 summarizes surgical outcomes. The main procedures performed were lobectomy in 59, segmentectomy in 24 and partial resection in 5 patients. The procedure was changed from the planned major lung resection to partial resection for 4 patients with an intraoperative diagnosis of noncancerous disease or metastatic lung tumour and 1 patient with noninvasive lung cancer and severe adhesions between the lung and chest wall. The median duration of surgery and console operation was 206 (126–368) and 157 (61–348) min, respectively. The median postoperative time of chest tube removal was 0 (0–7) days, and the duration of postoperative hospitalization was 3 (1–11) days. No serious postoperative complications occurred. One patient experienced a prolonged air leak (>5 postoperative days), and another patient showed worsening subcutaneous emphysema. The median duration of postoperative observation was 15.0 (1.5–28.3) months. One patient who underwent right lower lobectomy for pulmonary metastasis of glottic carcinoma with an uneventful postoperative course died unexpectedly 3 months postoperatively, probably from cardiovascular or cerebrovascular disease (details unknown). All other patients were doing well during the observation period. None of the patients experienced recurrence.

**Table 2:** Surgical outcomes of the 88 patients who underwent robotic lung resections using the three-arm, open-thoracotomy-view approach

Variables	Data^a^
Surgical procedure	
Lobectomy	59 (67)
RU/RM/RL/LU/LL	25/10/11/7/6
Segmentectomy	24 (27)
RU/RM/RL/LU/LL	4/0/5/12/3
Partial resection	5 (6)
RU/RM/RL/LU/LL	1/0/1/2/1
Operating time (median, range; min)	
Total time	206 (126–368)
Console time	157 (61–348)
Node dissection	
ND1/ND2a-1/ND2a-2	47 (53)/37 (42)/4 (5)
Bleeding (median, range; ml)	<5 (<5–440)
Number of stapling devices^b^ (median, range)	7 (2–16)
Conversion to open/to VATS	0/2
Morbidity	
Prolonged air leak (>5 postoperative days)	1 (1)
Subcutaneous emphysema	1 (1)
Paroxysmal atrial fibrillation	1 (1)
Postoperative course (median, range; days)	
Chest tube removal	0 (0–7)
Hospital stay	3 (1–11)
Histology	
Primary lung cancer	79 (90)
pTis/T1a/T1b/T1c/T2a/T2b/T3	3/26/33/9/7/0/1
pN0/N1/N2	78/1/0
p-Stage 0/IA1/IA2/IA3/IB/IIA/IIB	3/26/33/9/6/0/2
Metastatic lung tumour	5 (6)
Lymphoma	1 (1)
Other	3 (4)
Postoperative observation time (median, range; months)	15.0 (1.5–28.3)

a Data are presented as indicated or as the number of patients.

b Including robotic and nonrobotic devices.

LL: left lower; LU: left upper; RL: right lower; RM: right middle; RU: right upper; VATS: video-assisted thoracoscopic surgery.

No emergency or cool conversions to OTS occurred in all 109 patients who underwent RATS in our department (88 eligible patients for this survey who underwent RLRs with three-arm OTVA and 21 ineligible patients excluded from this study with different methods other than three-arm OTVA). Fortunately, no catastrophic events, such as a massive haemorrhage, occurred. However, 2 patients (2.3%, 2/88) needed unplanned conversions from the three-arm OTVA to regular confronting VATS. One patient underwent urgent conversion to VATS because of moderate Haemorrhage from a pulmonary artery branch during left upper lobectomy due to insufficient use of the robotic vessel sealing system in the introductive phase. A second patient, whose lung parenchyma was fragile with inflammatory changes, experienced a cool conversion to VATS during an extended right S6 + S10a segmentectomy, where robotic and nonrobotic staplers could not be inserted appropriately to resect the segmental boundary due to insufficient views and lung lacerations. The documents shown in the Figs. 1 and 2 helped us in both situations.

## DISCUSSION

Two of the total number of cases experienced by us so far have required VATS conversion. We are yet to experience an emergent open-thoracotomy conversion case. Thus, at this point, it may still not be sufficient to rigorously discuss our rollout procedures. Given the fact that such a critical situation requiring emergency thoracotomy is rare and cannot be necessarily experienced, that it may be too late to consider it after the event, and that the protocols presented here resulted in successful conversion in our VATS conversion cases, we consider that the procedures and discussions may be meaningful to some extent, although our experiences are at a preliminary and premature stage. We would like to emphasize the importance of careful team preparation for the emergency rollout and conversion procedures, even in the premature phase.

Cao *et al.* [2] reported that the conversion rate to OTS during RLRs was 7.1% (128/1810), and intraoperative catastrophic events occurred in 1.9% (35/1810) of patients based on a large, multicentre cohort investigation. Serious events, such as severe bleeding, were more frequent in patients with advanced disease or other difficult intrathoracic conditions. Our indications for RLR include early-stage lung cancer and/or relatively good intrathoracic conditions. Thus, we have experienced no severe events requiring OTS conversion to date.

In the conventional, look-up view approach for RLRs, 4 robotic ports and 1 assist port are around the caudal seventh or eighth intercostal spaces and 4 robotic arms are positioned over the patient. Therefore, in an emergency during the conventional approach, the patient’s ventral arm can be lifted to secure the space to convert to the OTS without rolling out the patient cart. In OTVA, alternatively, considering the port location and the position of the 3 robotic arms, the emergency rollout can be performed quicker than in the conventional setting. Therefore, from the viewpoint of obtaining a stable and sufficient thoracotomy conversion, we consider the complete rollout method better than the partial arm lifting method without rollout, even in emergency conditions for OTVA. In case of a catastrophic situation, thoracotomy conversion can be managed by lifting up the caudal 2 arms without rollout. In most cases, the thoracotomy is made at the intercostal space of the camera port. In right-side surgery, the assist port can be used for thoracotomy. In contrast, in the left-side surgery, the assist port may be difficult to use for thoracotomy because of its location at the caudal intercostal space. Whether to convert to OTS or VATS depends on the surgeon’s discretion; as a guide, we choose to convert to VATS when it is considered possible to manage the problem if a flexible camera movement can be secured because the camera manoeuvrability of confronting VATS is superior to that of robotic OTVA.

Cerfolio *et al.* [3] provided practical measures on managing unexpected bleeding, which are very valuable. In their report, the surgeon should try to remain calm and composed, and the arm lifting method without rollout is not recommended.

In our method, a human assistant B plays the role of the fourth arm, so our method is a hybrid method rather than a purely robotic method. Contrary to the trend towards future robotic surgeries performed by a single surgeon without assistants, our approach still requires 2 assistants. However, a human assistant acting as the fourth arm to complete a fine surgery is not necessarily disadvantageous. Having 2 assistants close to the patient also ensures safety in cases of emergency rollout and conversion to OTS or VATS.

Because our robotic OTVA is different from the most commonly used, well-established conventional look-up view approach, our method may be controversial and may present supporting and detracting perspectives. For RLRs, the widely-used, four-arm, look-up view method is undoubtedly considered as the current mainstream approach worldwide [6–9] including Japan [10]. On the other hand, some robotic surgeons certainly prefer OTVA. Yamazaki *et al.* [11] and Funai *et al.* [12] each described four-arm robotic approaches in which the patient’s craniocaudal direction can be viewed horizontally. In Yamazaki *et al.*’s anterior approach, the robotic instruments are always inserted from the ventral side of the patient’s thorax in both left- and right-side surgeries, and the left intrathoracic view is similar to that in our OTVA, whereas the right intrathoracic view is opposite to ours. Although these methods, including our own, are considered minor, this kind of approach is gradually being recognized. Similarly as VATS has several approaches including the look-up and CUD monitor methods, various approaches can be considered for RLR. Regardless of the robotic approach adopted, each member involved in the surgery should understand the roles and actions to be taken during an emergency. Therefore, we consider the presentation and discussion of emergency rollout procedures for our robotic OTVA essential.

### Limitations

The three-arm robotic OTVA method has several limitations, as described previously [1]. In addition, our experiences are still premature; further acquisition of experience is necessary. A retrospective analysis of data from a single institution also limits the generalization of the methodology and the findings.

## CONCLUSION

The three-arm OTVA using the vertical port placement and CUD monitor setting, which allows us to actualize natural thoracotomy views, is an option for RLR. In an emergency, the rollout and conversion procedures indicated in Figs. 1 and 2 are possible actions. Things may not go according to plan in an emergency. However, regardless of which robotic approach is adopted, careful preparation and awareness of the points to be considered in each approach are crucial to promote RATS safely.

**Conflict of interest:** none declared.

## Data Availability

All relevant data are within the manuscript.

## ABBREVIATIONS

CUD: Confronting upside-down
OTS: Open-thoracotomy surgery
OTVA: Open-thoracotomy-view approach
RATS: Robot-assisted thoracoscopic surgery
RLR: Robotic lung resection
VATS: Video-assisted thoracoscopic surgery
VMST: Vertical muscle-sparing/splitting thoracotomy
